# The natural history and burden of illness of metachromatic leukodystrophy: a systematic literature review

**DOI:** 10.1186/s40001-024-01771-1

**Published:** 2024-03-18

**Authors:** Shun-Chiao Chang, Christian Stefan Eichinger, Polly Field

**Affiliations:** 1 Takeda Development Center Americas, Inc., 125 Binney Street, Cambridge, MA USA; 2 Oxford PharmaGenesis, Oxford, UK

**Keywords:** Metachromatic leukodystrophy, MLD, Natural history, Burden of illness, Systematic literature review, Lysosomal storage disease

## Abstract

**Background:**

Metachromatic leukodystrophy (MLD; OMIM 250100 and 249900) is a rare lysosomal storage disease caused by deficient arylsulfatase A activity, leading to accumulation of sulfatides in the nervous system. This systematic literature review aimed to explore the effect of MLD on the lives of patients.

**Methods:**

The Ovid platform was used to search Embase, MEDLINE, and the Cochrane Library for articles related to the natural history, clinical outcomes, and burden of illness of MLD; congress and hand searches were performed using ‘metachromatic leukodystrophy’ as a keyword. Of the 531 publications identified, 120 were included for data extraction following screening. A subset of findings from studies relating to MLD natural history and burden of illness (*n* = 108) are presented here.

**Results:**

The mean age at symptom onset was generally 16–18 months for late-infantile MLD and 6–10 years for juvenile MLD. Age at diagnosis and time to diagnosis varied widely. Typically, patients with late-infantile MLD presented predominantly with motor symptoms and developmental delay; patients with juvenile MLD presented with motor, cognitive, and behavioral symptoms; and patients with adult MLD presented with cognitive symptoms and psychiatric and mood disorders. Patients with late-infantile MLD had more rapid decline of motor function over time and lower survival than patients with juvenile MLD. Commonly reported comorbidities/complications included ataxia, epilepsy, gallbladder abnormalities, incontinence, neuropathy, and seizures.

**Conclusions:**

Epidemiology of MLD by geographic regions, quantitative cognitive data, data on the differences between early- and late-juvenile MLD, and humanistic or economic outcomes were limited. Further studies on clinical, humanistic (i.e., quality of life), and economic outcomes are needed to help inform healthcare decisions for patients with MLD.

**Supplementary Information:**

The online version contains supplementary material available at 10.1186/s40001-024-01771-1.

## Background

Metachromatic leukodystrophy (MLD; OMIM 250100 and 249900) is a rare, life-limiting lysosomal storage disease (LSD). It is caused by pathogenic variants in the arylsulfatase A (ASA) or sphingolipid activator protein B (SAPB) genes (*ARSA* and *PSAP*, respectively), which are inherited in an autosomal recessive manner [1, 2]. Approximately 261 variants of *ARSA* and 64 variants of *PSAP* have been reported previously, and the majority of cases of MLD are associated with ASA deficiency [1, 2]. ASA or SAPB deficiency leads to accumulation of sulfatides throughout the body and is particularly detrimental to nervous system function [3]. The incidence (birth prevalence) of MLD varies across populations but has been estimated to be between 1 in 40,000 and 1 in 160,000 [1].

There are three clinical subtypes of MLD, based on the age at first symptom onset: late-infantile (onset ≤ 2.5 years), juvenile (onset 2.5– < 16 years), and adult (onset ≥ 16 years) MLD [4, 5]. Juvenile MLD has been further subdivided into early-juvenile and late-juvenile forms, which have onsets before or after the age of 6 years, respectively [5]. There is some evidence that disease onset with motor symptoms is associated with a more rapid disease progression than onset with cognitive symptoms only [5]. Patients with MLD are substantially impacted by a wide range of signs and symptoms, including gait abnormalities, speech regression, and seizures [1, 4]. Across all clinical subtypes of MLD, the lives of patients are shortened, with many patients not reaching adulthood [3, 4].

Published evidence about the natural history of MLD and how the disease affects the lives of patients is limited and has not, to our knowledge, been reviewed systematically.

For this systematic literature review (SLR), our aim was to understand how MLD affects the lives of patients. Here, we report a subset of our findings, focused on the epidemiology and natural history of MLD, including disease progression and associations between physical function and disease progression. While the focus of this SLR was on patients with late-infantile or juvenile MLD, relevant information from patients with adult MLD was also considered.

## Methods

### Search strategy

This study was conducted in accordance with guidelines from the Cochrane Collaboration [6], the University of York Centre for Reviews and Dissemination (CRD) [7], and the Preferred Reporting Items for Systematic review and Meta-Analysis (PRISMA) [8], and it was registered in the PROSPERO database.

The Ovid platform was used to search Embase (1974–2021), MEDLINE, and the Cochrane Library on June 23, 2021. The search strings used (Additional files 1, 2, 3) related to the natural history, clinical outcomes (not reported here), and burden of illness of MLD for patients with MLD. Congress and hand searches were also performed using ‘metachromatic leukodystrophy’ as a keyword. Congress searches covered the following congresses from 2020 to 2021: WORLD*Symposium*; International Congress of Inborn Errors of Metabolism; Society for the Study of Inborn Errors of Metabolism; International Society of Pharmacoeconomics and Outcomes Research European, US, and international congresses; Society for Inherited Metabolic Disorders; Child Neurology Society; American Neurological Association; European Society of Human Genetics; European Paediatric Neurology Society; European Academy of Neurology; American College of Medical Genetics and Genomics; European Society of Gene and Cell Therapy; and Congress of Neurological Surgeons. Hand searches covered the websites for ClinicalTrials.gov, the Cost-Effectiveness Analysis Registry, the International Clinical Trials Registry Platform, Research Papers in Economics, the University of Sheffield School of Health and Related Research Health Utilities Database, and the University of York CRD.

### Eligibility criteria

The Participants, Interventions, Comparators, Outcomes, and Study design (PICOS) criteria are summarized in Table 1. For study titles and abstracts, double-blind screening was performed by two researchers, and any uncertainties were checked by a third reviewer. For congress abstracts and hand searches, screening was performed by one reviewer.

**Table 1:** PICOS eligibility criteria

Eligibility criteria	Inclusion criteria
Population	Patients with MLD^a^ • Late-infantile MLD • Juvenile MLD • Adult MLD
Interventions	Any or none
Comparators	Any or none
Outcomes	Natural history evidence • Association between GMFC-MLD at baseline or phenotype and outcomes in the future, especially progression-related • To include evidence for which an association has already been tested and evidence that could later be used for statistical testing (e.g., any longitudinal data) • Specific question: does treatment^b^ give longer time in a more severe disease state? Clinical outcomes • Treatment options (best supportive care, HSCT, gene therapy, etc.), associated clinical outcomes in different disease stages, and variability across key markets •Disease progression (including but not limited to): • gross motor function • cognitive function • difficulty in eating and drinking • difficulty in breathing • Morbidity and mortality associated with different treatment options stratified by: • clinical subtype (late-infantile, juvenile, or adult MLD) • disease stages • time period • Treatment efficacy and/or effectiveness, treatment safety • Response and change from baseline evaluated using GMFC-MLD, including time to unreversed decline • Response and change from baseline evaluated using GMFM-88, including total score decline • Change from baseline in expressive language evaluated using ELFC-MLD • Change from baseline in CSF sulfatide levels • Change from baseline in proton MRS metabolite level of *N*-acetylaspartate/creatine • Change from baseline in Eichler MLD MRI severity score • TEAEs • AEs (grade > 3) • Pharmacokinetic measurements • HRQoL and patient-reported outcomes (LQLA; Vineland Adaptive Behavior Scales; PedsQL Family Impact Module, EQ-5D-5L, and EQ-5D-Y), COMFORT •Association between benefit for patient subgroups and types of treatments (especially HSCT) • Humanistic and economic burden of illness evidence • Healthcare resource use and costs (by clinical subtype and phenotype if reported) •Direct healthcare-related resource use (e.g., number of hospital admissions, days per admission) • Cost of treatments • Indirect healthcare cost (e.g., home modifications, wheelchairs, transportation, cost of care) • Societal resource use (e.g., days that the caregivers take off work, percentage of people who quit their jobs) •Economic evaluations • Quality-adjusted life-years gained •Progression-free life-years gained • Life-years gained •Health state utilities • Treatment patterns by geography (especially use of HSCT)
Study design	Natural history • Real-world observational/non-interventional studies • Clinical evidence • RCTs, single-arm trials and real-world observational/non-interventional studies • Humanistic and economic burden of illness evidence • Not limited by study type • All evidence • SLRs and meta-analyses^c^ • Animal/in vitro studies and case reports will be excluded; case series will be included
Date restrictions	•No limit
Publication type	• All primary publications and SLRs^c^ • Non-SLRs, editorials, notes, and letters will be excluded
Country and language	• All countries if English language

AE: adverse event; COMFORT: Caregiver Observed MLD Functioning and Outcomes Reporting Tool; CSF: cerebrospinal fluid; ELFC-MLD, Expressive Language Function Classification in MLD; EQ-5D-5L: EuroQoL 5-dimension 5-level; EQ-5D-Y: EuroQoL 5-dimension youth; GMFC-MLD: Gross Motor Function Classification in MLD;

GMFM-88: Gross Motor Function Measure 88-item; HRQoL: health-related quality of life; HSCT: hematopoietic stem cell transplantation; LQLA: Leukodystrophy Quality of Life Assessment; MLD: metachromatic leukodystrophy; MRI: magnetic resonance imaging; MRS: magnetic resonance spectroscopy; PedsQL: Pediatric Quality of Life Inventory; PICOS: Participants, Interventions, Comparators, Outcomes, and Study design; RCT: randomized controlled trial; SLR: systematic literature review; TEAE: treatment-emergent adverse event

^a^Studies reporting adult populations were initially tagged at full-text review without data extraction

^b^Best supportive care

^c^The reference lists from these publications were cross-checked against lists of included references in our SLR to ensure that all relevant data had been identified.

Any additional relevant data were extracted

### Full-text review and data extraction

Single-blind full-text review was performed by one reviewer, with a second person resolving any uncertainties to confirm eligibility. Data were extracted into a predefined data extraction table by one reviewer and independently checked for errors by a second reviewer. Discrepancies were resolved through discussion or with the intervention of a third reviewer. For papers that reported data across LSDs or on leukodystrophy cohorts, data were extracted for the patients with MLD only if outcomes were reported separately for these patients.

### Quality assessment

No randomized controlled trials were identified, but quality assessment using the Risk Of Bias In Non-randomized Studies - of Interventions (ROBINS-I) tool was completed for all full publications reporting data from non-randomized interventional clinical studies. For publications reporting data from non-interventional studies, the overall quality of the evidence identified was informally assessed based on a review of study methods and population size.

## Results

### Search results

**Fig. 1:**
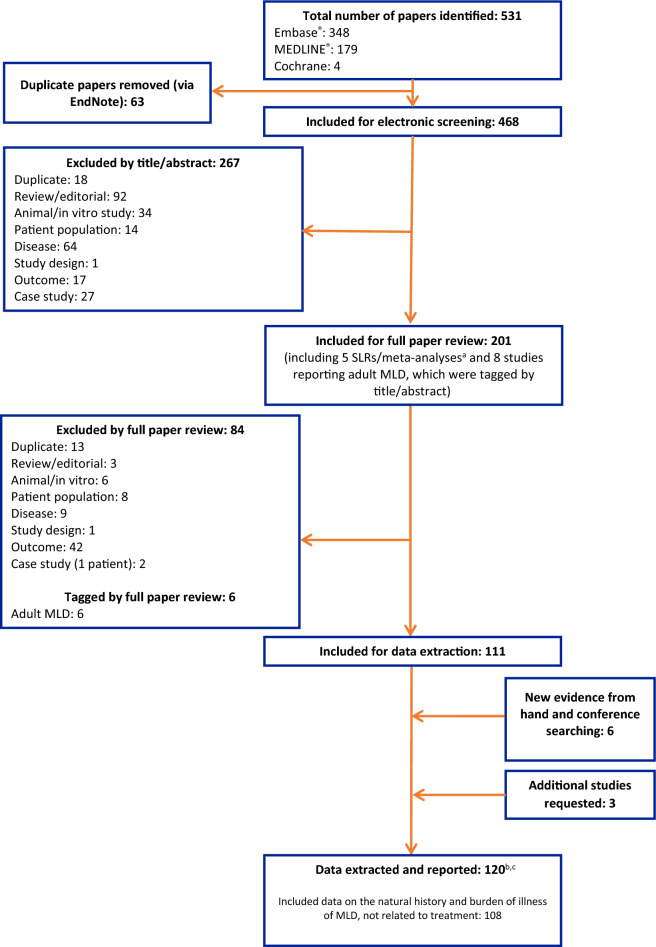
PRISMA diagram. ^a^The reference lists from these publications were cross-checked against lists of included references to ensure that all relevant data were identified. Data from SLRs/meta-analyses were not extracted. ^b^Four references were data-extracted, but data were either not reported separately for patients with MLD, or no relevant data were reported in the publication. ^c^Of the 120 publications from which data were extracted, 108 related to the natural history and burden of illness of MLD (i.e., non-treatment related). MLD: metachromatic leukodystrophy; PRISMA: Preferred Reporting Items for Systematic review and Meta-Analysis; SLR: systematic literature review

The search identified 531 publications, of which 63 were removed as duplicates before title/abstract screening and 267 were excluded following title/abstract screening (Fig. 1). The full texts of the remaining 201 publications were screened against the PICOS criteria, following which 111 published studies were considered eligible for inclusion. Six abstracts were also identified from the congress searches, and three studies were included on request. In total, data were extracted from 120 studies, of which 88 were full publications and 32 were abstracts. Of the 120 studies, 22 were interventional in design (all of which were single-armed) and the remaining 98 were observational. All non-randomized, interventional studies were assessed with the ROBINS-I tool and determined to be of moderate quality.

The studies selected for data extraction used patient data from countries across six continents. The countries with the largest number of studies reporting patient data were the USA, Germany, and Italy (20, 12, and 12 studies, respectively). Six studies included data from multiple countries/continents, and the country was not reported or unclear for 13 studies. We report a subset of studies (*n* = 108) that describe the natural history and burden of illness of MLD. Of these, 70 were natural history studies. Note that any data from the remaining observational, retrospective, or interventional studies (*n* = 38) were related to patients who were not receiving treatment in the form of hematopoietic stem cell transplantation, gene therapy, or enzyme replacement therapy, except where otherwise stated.

### Epidemiology

The birth incidence and birth prevalence of MLD were reported in eight [9–16] and four [13, 14, 16, 17] studies, respectively (Table 2). For birth incidence, only postnatal diagnoses were considered, whereas for birth prevalence, both prenatal and postnatal diagnoses were considered. The data from these studies spanned 10 countries over a wide time frame and used different methodologies. 

The Czech Republic was reported to have the lowest birth prevalence of MLD at 0.69 per 100,000 births [16], and Poland was reported as having the highest birth prevalence of 4.1 per 100,000 births [13]. Sweden was reported to have the highest incidence of MLD at 2.5 per 100,000 births [10], and Japan was reported to have the lowest incidence at 0.16 per 100,000 births [16].

**Table 2:** Birth incidence and birth prevalence of MLD

Author(s), year country	Time period	Birth incidence of MLD	Birth prevalence of MLD	Country/region reported	Number of diagnosed cases of MLD	Total population
Artigalas et al*.* 2010 [9] Brazil	2003–2007	Minimum incidence of MLD: 0.67 per 100,000 live births	NR	Rio Grande do Sul, Brazil	5	745,971 live births
Gustavson and Hagberg. 1971 [10] Sweden	1955–1965	Birth incidence for late-infantile MLD: ~ 1 per 40,000 births (2.5 per 100,000 births)^a^	NR	Umeh and Uppsala, Sweden	8 late-infantile; 1 juvenile	316,786 total births
Heim et al. 1997 [11] Germany	1984–1990	Minimal incidence of all MLD subtypes of both sexes: 0.6 per 100,000 live births	NR	Germany	41	NR
Hult et al. 2014 [12] Sweden	1980–2009	Incidence of MLD: 1.73 diagnoses per 100,000 births, corresponding to 1 per 58,000 births	NR	Sweden	36	2,080,791 births
Koto et al. 2021 [16]^,b^ Japan	1975–2013	Birth incidence of MLD: 0.16 per 100,000^c^	NR	Japan	83 (estimated number of patients)	NR
	NR	NR	1.09 per 100,000	Australia (Meikle et al. 1999) [14]	NR	NR
			1.42 per 100,000	Netherlands (Poorthuis et al. 1999) [17]	NR	NR
			1.85 per 100,000	Portugal	NR	NR
			0.69 per 100,000	Czech Republic	NR	NR
			1.43 per 100,000^d^	Turkey	NR	NR
			2.0 per 100,000	USA (Bonkowsky et al. 2018) [123]	NR	NR
Lugowska et al. 2011 [13] Poland	1975–2004 1960–2009	Estimated incidence: 0.38 per 100,000 live births based on diagnosed cases	Expected birth prevalence (based on carrier rates)^e^ per 100,000 conceived fetuses • Cohort 1: 4.0 (95% CI: 1.7–9.6), or 1 in 25,000 • Cohort 2: 4.1 (95% CI: 1.4–12.4), or 1 in 24,390 • Pooled estimate: 4.1 (95% CI: 1.8–9.4), or 1 in 24,390 with two pathogenic mutations	Poland	62 (1975–2004) 73 (1960–2009)	16,332,700 (number of births between 1975 and 2004) 26,895,000 (number of births between 1960 and 2009)
Meikle et al. 1999 [14] Australia	January 1980 to December 1996	Incidence in 1000s:^f^ 121 (1/121,000 = 0.83 per 100,000 births)^a^	Prevalence in 1000s:^f^ 92 (1/92 000 = 1.09 per 100,000 births)^a^	Australia	46 (35 postnatal, 11 prenatal)	545 lysosomal storage disease diagnoses
Poorthuis et al. 1999 [17] The Netherlands	• Late-infantile: 1965–1991^g^ • Juvenile: 1954–1991^g^ • Adult: 1927–1970^g^ • Unspecified: (1957–1992)^g^	NR	Birth prevalence per 100,000: • late-infantile: 0.52 • juvenile: 0.51 • adult: 0.24 • unspecified: 0.15 all: 1.42	Netherlands	• Late-infantile: 28 • Juvenile: 41 • Adult: 23 • Unspecified: 11 • All: 103	Number of live births: • Late-infantile: 5,346,384 (1965–1991) • Juvenile: 7,982,018 (1954–1991) • Adult: 9,517,068 (1927–1970) • Unspecified: 7,489,865 (1957–1992)
Stellitano et al. 2016 [15] UK	1997–2014	Estimated lifetime risk per million UK live births: 5.8^g^ (0.58 per 100,000 live births)^a^	NR	UK	76	3758 notifications of children meeting criteria for progressive intellectual and neurological deterioration; diagnosed leukodystrophies *n* = 349
Zlotogora et al. 2016 [94] Israel	2013–2014	NR	NR	Israel	9 carriers^h,i^	891 Yemenite Jews

*ARSA*: arylsulfatase A gene; CI: confidence interval; MLD: metachromatic leukodystrophy; NR: not reported

^a^Converted for ease of interpretation

^b^Data from the Netherlands and Portugal only included patients with an enzymatic diagnosis, while the report from Turkey included patients younger than 5 years of age. Therefore, the number of patients may be underestimated

^c^These data were reported in the original publication as birth prevalence, but because they do not include prenatal data, they have been reported here as birth incidence

^d^These data were reported in the original publication as birth incidence, but because they include prenatal data, they have been reported here as birth prevalence

^e^Based on mutation carrier rates of the *ARSA* gene for cohort 1: c.459 + 1G > A, p.P426L, and p.I179S among individuals undergoing paternity testing; cohort 2: c.459 + 1G > A and p.I179S in a study population representative of the Polish population; and pooled cohort: c.459 + 1G > A and p.I179S

^f^Incidence was calculated by dividing the number of postnatal diagnoses by the number of births during the study period

^g^Born during the indicated period

^h^Corrected in 2017 corrigendum[15]

^i^By genetic screening of newborn children for the P377L mutation of the *ARSA* gene

The percentage of MLD cases within leukodystrophies was reported in nine studies [11, 18–25] and ranged from 8.0% (country not reported) [18] to 42.4% (Saudi Arabia) [22]. One study reported a proportion lower than this range (3.0%), but it only included individuals who did not have diagnostic testing in another healthcare system and who had no previous family members with the same diagnosis [25]. The reported proportion of MLD within LSDs was reported in seven studies [12, 14, 16, 26–29] and ranged from 3.3% (Japan) to 47.6% (Tunisia); however, the inclusion criteria varied between the studies. Those reporting a higher proportion of MLD within leukodystrophies or LSDs tended to have a smaller study population or were conducted in countries with higher rates of consanguinity. Three studies reported the proportion of MLD in inborn errors of metabolism, which ranged from 1.4% to 18.2% [29–31]. Three studies reported the prevalence of MLD in lipidoses, which ranged from 18.0% to 30.2% [12, 17, 32].

### Natural history

Age at symptom onset was reported in 51 studies [5, 9, 10, 22, 24, 32–77]. Across all studies, the range of ages at symptom onset (for individual patients) was 0.5–3 years for late-infantile MLD [9, 10, 34, 35, 38, 44, 45, 49–51, 53, 55, 57–59, 61–65, 72, 75, 77], 2–16 years for juvenile MLD (one study exclusively recruited early-symptomatic, early-juvenile patients) [9, 10, 34, 35, 45, 47, 49, 51–53, 55, 57, 63–65, 71, 72, 75, 77, 78], and 17–35 years for adult MLD [49, 72]. In most studies, mean age at symptom onset ranged from 16 to 18 months for the late-infantile subtype of MLD. For juvenile MLD, mean age at symptom onset ranged from 6 to 10 years [5, 9, 10, 22, 24, 32–77].

Age at diagnosis was reported in 19 studies [9, 12, 16, 22, 35, 39, 42, 44, 53, 60, 66, 72, 73, 79–84], of which seven [9, 53, 66, 79, 81–83] reported data for the different clinical subtypes of MLD. The age at diagnosis ranged from 0.4 to 8.6 years for late-infantile MLD [9, 12, 14, 16, 35, 44, 53, 72, 79, 81–84], from 3.0 to 21.6 years for juvenile MLD [9, 12, 14, 16, 35, 53, 72, 81, 82, 84], and from 17.0 to 35.3 years for adult MLD [72, 84]. Five of these studies reported children who received a diagnosis after an affected sibling [39, 42, 53, 72, 80].

Time to diagnosis from onset of symptoms was reported in 11 studies [9, 22, 38, 39, 53, 57, 69, 81, 85–87]. Based on five studies that reported MLD-subtype-specific data, the ranges for time to diagnosis from symptom onset were 0–7.1 years for late-infantile MLD, with one study that reported a range from “almost immediately” to 13 months [81], and 0.1–23.5 years for juvenile MLD [9, 53, 57, 81, 85]. It should be noted that some of the studies reporting data on age at symptom onset and age at diagnosis did not report data for untreated patients specifically; therefore, some of the patients may have received treatment during or before the study.

### Genetic variants

Although genetic variation was not a prespecified outcome of interest in this review (see Table 1), any genotype information in the identified studies was recorded and is later summarized. *ARSA* variants associated with MLD were reported in 27 studies [5, 13, 15, 22, 24, 45, 49, 50, 52, 60–62, 66, 67, 69, 72, 75, 76, 80, 84, 88–94]. Most of these studies reported genetic variants per patient as a case series. Lugowska et al. [90] reported the distribution of two variants by MLD subtype in a population from 16 European countries. The c.459 + 1G > A variant was found at a rate of 25% in patients with MLD (194/768 patients) and was found more commonly in patients with late-infantile MLD (137/344 [40%]) than in other clinical subtypes of MLD (juvenile: 36/222 [16%]; adult: 15/160 [9%]). The p.P426L variant was found at a rate of 18.6% in patients with MLD (143/768 patients) and was considerably more common in juvenile MLD (66/222 [30%]) and adult MLD (68/160 [42.5%]) than in late-infantile MLD (3/344 [0.9%]). Note that some of these studies did not report data for untreated patients specifically, so some patients may have received treatment during or before the study.

### Initial symptoms in untreated children

Qualitative descriptions of disease severity at baseline in untreated children were identified in 20 studies, all of which were observational studies [5, 9, 24, 34, 35, 38, 43, 45, 56, 57, 62, 65, 71, 72, 74, 75, 89, 92, 95, 96]. Patients with late-infantile MLD generally presented predominantly with motor symptoms and developmental delays, whereas patients with juvenile MLD generally presented with motor, cognitive, and behavioral symptoms. The initial presentation of adult MLD was typified by cognitive symptoms and psychiatric and mood disorders.

Thirteen studies reported differences in initial symptoms between the late-infantile and juvenile forms of MLD [5, 9, 24, 34, 35, 38, 49, 53, 57, 63, 65, 81, 86]. The findings of these studies are summarized in Table 3. Patients with early-juvenile MLD were more likely to have some level of motor impairment with cognitive deficits, whereas patients with late-juvenile MLD were more likely to have predominantly cognitive or behavioral symptoms [5, 34, 49].

**Table 3:** Comparison of initial symptoms reported in late-infantile and juvenile MLD

Author(s), year country	Numbers of patients				Initial symptoms, number or %	
	MLD total	Late-infantile MLD	Juvenile MLD	Adult MLD	Late-infantile MLD	Juvenile MLD
Artigalas et al. 2010 [9] Brazil	29	22	4	1	• Gait abnormality or frequent falls: 72.7% • Neuropsychomotor development delay: 31.8% • Behavioral abnormalities: 4.5% • Cognitive deficit: 4.5%	• Cognitive deficit associated with walking alterations: 25% • Cognitive deficit: 50% • Behavioral and cognitive alterations: 75%
Bascou et al. 2020 [35] Unclear	122	NR	NR	NR	Patients with onset before 3 years of age presented predominantly with gross motor involvement	Patients with onset between 6 and 16 years of age presented mainly with cognitive impairment
Bascou et al. 2017 [34] USA	104	63^a^	• Early-juvenile: 22^b^ • Late-juvenile: 14^b^	5^b^	• Delayed achievement of gross motor milestones: 46.0% • Abnormal gait: 28.6% • Gross motor regression: 20.6%	• Early-juvenile MLD had similar initial symptoms to late-infantile MLD • Some cases involved impairment of fine motor skills and language acquisition • Late-juvenile MLD had mainly cognitive deficiencies • Memory/attention/learning difficulties: 50% • Changes in personality: 21.4%
Bindu et al. 2005 [38] India	40	36	4	0	• Baseline delay in milestones followed by regression: 38.8% (14/36) – Others developed regression after a period of normal development • Independent walking attained before the onset of illness: 50% (18/36) – Frequent falls as the initial symptom: 78% (14/18)	• Behavioral abnormalities such as attention deficits, hyperactivity, inappropriate laughter, and hyper-oral behavior: 100% (4/4) • Learning problems and scholastic backwardness: 100% (4/4) • Myoclonic jerks: 75% (3/4) • Sluggish or absent deep tendon reflexes, indicating underlying neuropathy: 100% (4/4)
Carson et al. 2015 [86] USA	90	58	29	3	Initial symptoms were mostly related to changes in gait	In late-juvenile MLD, the initial symptoms were changes in cognitive and behavioral function
Eichler et al. 2016 [81] USA, France, Germany, and Colombia	23	14	6	3	Initial symptoms included: • frequent falling or other walking problems • regression of speech or motor skills • behavioral abnormalities	Initial symptoms included: • gait problems • behavioral abnormalities
Fumagalli et al. 2021 [49] Italy	45	22	19 Early-juvenile: 14 Late-juvenile: 5	4	• No independent walking: 40.9% (9/22) • No independent sitting: 4.5% (1/22) • Gait impairment (unsteadiness, frequent falls, and toe walking variably associated with musculoskeletal abnormalities such as foot deformities or retro-curved knees): 54.5% (12/22) • Nystagmus: 4.5% (1/22) • Strabismus: 9.1% (2/22)	Early-juvenile MLD • Isolated gross motor impairment (tripping or falling, clumsiness, and poor balance): 28.6% (4/14) • Isolated behavioral and/or cognitive regression: 35.7% (5/14) • Decline of both cognitive and fine or gross motor skills: 35.7% (5/14) • Seizures: 7.1% (1/14) • Loss of bladder control: 35.7% (5/14) • Late-juvenile MLD • Isolated behavioral and cognitive regression, such as attention deficit, reduced school performance, social withdrawal, and loss of sphincter control: 100% (5/5)
Harrington et al. 2019 [53] USA	32 plus eight siblings	16	16	0	• Problems with gross motor function (particularly in walking): 75.0% (12/16) • Never learned to walk independently: 68.8% (11/16) • Experienced symptoms relating to gross motor function by the time of diagnosis: 93.8% (15/16) • Decline in cognitive function before diagnosis: 6.3% (1/16)	• Changes in cognitive function: 56.3% (9/16) • Changes in social/behavioral function: 43.8% (7/16) • Decline in gross motor function by the time of diagnosis (slowed movements, affected gait, and loss of balance): 56.3% (9/16)
Kehrer et al. 2014 [57] Germany	59	23	36	0	None of the patients with late-infantile MLD had exclusively “non gross motor” symptoms as the first signs of disease (*P* values are for late-infantile vs. juvenile MLD) • Gait disturbance: 70% (16/23); *P* = 0.9922 • Pain: 26% (6/23); *P* = 0.2447 • Abnormal movement patterns: 61% (14/23); *P* = 0.5011 • Impaired fine motor skills: 17% (4/23); *P* = 0.0011 • Restlessness/irritability: 17% (4/23); *P* = 0.3645 • Weakness: 43% (10/23); *P* = 0.5748 • Problems with concentration: 0% (0/23); *P* = 0.0001 • Behavioral problems: 13% (3/23); *P* = 0.0012 • Developmental regression in general: 61% (14/23); *P* = 0.5449	• Exclusively “non-gross-motor” symptoms as the first signs of disease: 17% (6/36) • “Impaired fine motor skills”, “concentration problems”, and “behavioral problems”: 36% (13/36) • Gait disturbance: 69% (25/36) Pain: 14% (5/36) • Abnormal movement patterns: 69% (25/36) • Impaired fine motor skills: 61% (22/36) • Restlessness/irritability: 28% (10/36) • Weakness: 36% (13/36) • Problems with concentration: 64% (23/36) • Behavioral problems: 56% (20/36) • Developmental regression in general: 53% (19/36)
Kehrer et al. 2021 [5] Germany	97	35	56 • Early-juvenile: 18 • Late-juvenile: 38	6	• Motor symptoms only: 91% (32/35) • Motor and cognitive symptoms: 9% (3/35)	Early-juvenile MLD • Motor symptoms only: 61% (11/18) • Motor and cognitive symptoms: 39% (7/18) • Late-juvenile MLD • Cognitive symptoms only: 61% (23/38) • Motor symptoms only: 13% (5/38) • Motor and cognitive symptoms: 26% (10/38)
Mahmood et al. 2010 [63] USA	303 (SLR) 3 cases	98 (SLR) 3 cases	78 (SLR)	127 (SLR)	A total of 38 late-infantile cases had detailed clinical features provided • Motor or gait abnormalities: 61% Seizures: 39%	The first symptom was rapid progression in lower extremity muscle tone; all three siblings lost the ability to walk within days of each other • Pooled SLR survival analysis • Inattention and difficulties at school: 66% • Gait difficulties: 26% • Tremor or ataxia: 18% • Neuropathy: 13% • Seizures: 5%
Raina et al. 2019 [65] India	12	4	8	0	Reported symptoms at presentation^b^ • Motor impairment: 100% (4/4) • Neurocognitive impairment: 100% (4/4) • Language impairment: 100% (4/4) • Seizures: 25% (1/4) • Psychomotor regression: 75% (3/4) • Bilateral spasticity: 100% (4/4) • Areflexia: 50% (2/4)	Reported symptoms at presentation^b^ • Motor impairment: 75% (6/8) • Neurocognitive impairment: 87.5% (7/8) • Language impairment: 87.5% (7/8) • Seizures: 12.5% (1/8) • Psychomotor regression: 0% (0/8) • Gait difficulty: 63% (5/8) • Extrapyramidal features (generalized dystonia): 38% (3/8) • Ataxia: 25% (2/8)
Saeed et al. 2017 [24] Saudi Arabia	14	12	2	NR	All presented with psychomotor regression of achieved developmental milestones (motor milestones regression, cognitive deterioration, and generalized spasticity) Other clinical findings • Optic atrophy: 100% (14/14) • Convulsions: 14.25% (2/14) • Small head size: 14.25% (2/14)	
						The two patients who received a diagnosis of juvenile MLD presented with ataxia and cognitive decline

MLD: metachromatic leukodystrophy; NR: not reported; SLR: systematic literature review

^a^Percentages reported in the study have been converted to *n* numbers for ease of comparison

^b^Patients were subclassified into late-infantile and juvenile MLD based on the age of onset of symptoms and their clinical characteristics

The most frequently reported initial symptoms affecting motor function were gait disturbances, walking difficulties, frequent falls, and problems with balance, and the most frequently reported symptoms affecting cognitive function included learning difficulties, language acquisition, and behavioral changes. Other frequently reported initial symptoms were neuropathy, ataxia, seizures, tremor, and incontinence.

### Gross Motor Function Classification in MLD (GMFC-MLD) level at baseline

The GMFC-MLD is a clinician-rated classification system of motor decline in MLD [97]. It has seven categories representing clinically relevant stages from normal (category 0) to loss of all locomotion (category 6) including head and trunk control [97]. GMFC-MLD level at disease baseline in untreated patients was reported in five studies, which are later described in detail [50, 56, 65, 72, 85]. Unless stated otherwise, baseline was defined as the patient’s GMFC-MLD level at the time of first assessment as reported by each study. There was a trend for patients with late-infantile MLD to have a higher baseline GMFC-MLD level than patients with juvenile or adult MLD.

Kehrer et al. reported GMFC-MLD levels in patients at the age of 18 months, which is the earliest age at which the scale can be reliably used. Gross motor function before 18 months was considered ‘normal’ if there was no evidence of motor regression and if developmental milestones were achieved at the expected chronological age. There was a significant difference between late-infantile and juvenile MLD; only 5/28 patients with late-infantile MLD had ‘normal’ gross motor function before the age of 18 months, whereas all patients with juvenile MLD had ‘normal’ motor function at this age (*P* < 0.001). At age 18 months, most patients with late-infantile MLD were at GMFC-MLD level 1 or 2 (level 0: *n* = 3; level 1: *n* = 7; level 2: *n* = 9; level 3: *n* = 3), whereas all patients with juvenile MLD were at level 0 [56].

Raina et al. retrospectively reported median GMFC-MLD levels in patients’ first recorded clinical, electroneurography, and neuroimaging examinations. Patients with late-infantile MLD tended to be at a higher level of GMFC-MLD than patients with juvenile MLD (late-infantile MLD [*n* = 4]: level 6 [range: 2–6]; juvenile MLD [*n* = 8]: level 2 [1–6]) [65].

Tillema et al. collected data on GMFC-MLD levels via a retrospective clinical chart review as part of a study in patients who had undergone magnetic resonance imaging (MRI) scans shortly after diagnosis. The study reported a median GMFC-MLD score of 1 (range: 0–6) for childhood-onset MLD (late-infantile MLD: *n* = 3; juvenile MLD: *n* = 11) and a median score of 0 (range: 0–1) for adult MLD (*n* = 6) [72].

Groeschel et al. reported cerebral volumetric changes as assessed by MRI in patients with late-infantile MLD (*n* = 18) and compared these cross-sectionally with those from typically developing children in the same age range (*n* = 42). The GMFC-MLD level for each patient with MLD at the time of MRI examination was reported; most patients were at the higher levels of GMFC-MLD 

(level 1: *n* = 1; level 2: *n* = 4; level 3: *n* = 1; level 4: *n* = 1; level 5: *n* = 4; level 6: *n* = 7) [50].

Ammann-Schnell et al. reported the results of semi-standardized questionnaires completed by the parents of patients with MLD. At the time of the survey, parents reported a median GMFC-MLD level of 6 (range: 5–6) for patients with late-infantile MLD (*n* = 8) and 4.5 (0–6) for patients with juvenile MLD (*n* = 18) [85].

### Comorbidities and complications

**Fig. 2:**
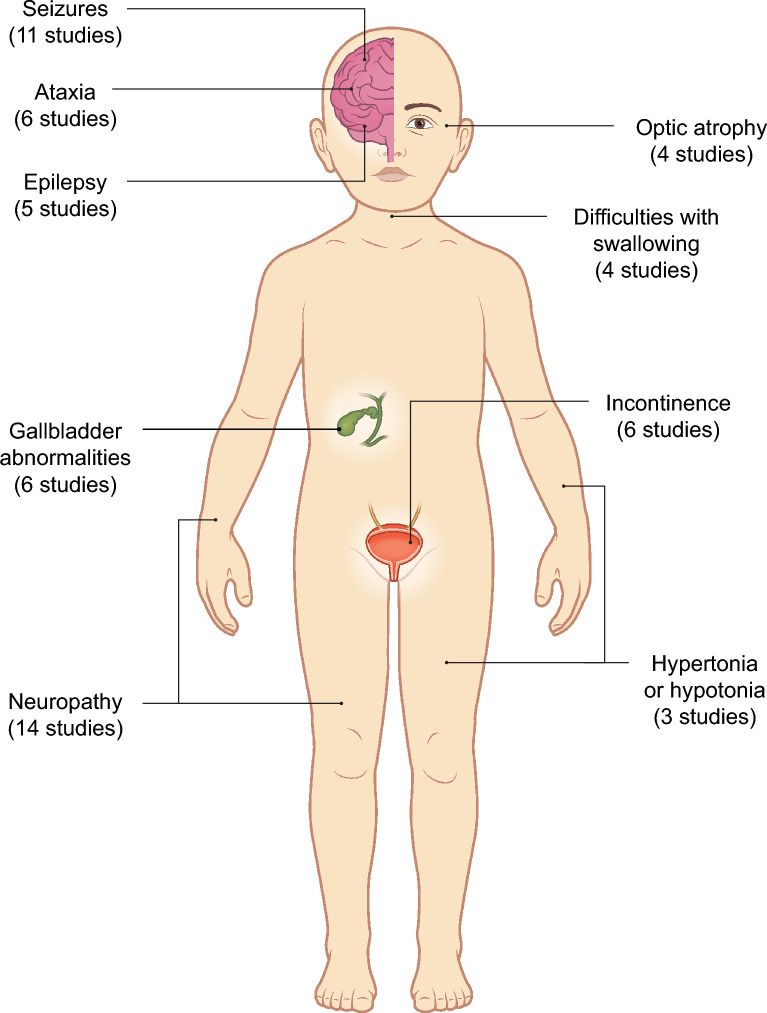
MLD comorbidities and complications reported in three or more studies. Comorbidities and complications related to MLD were reported in 35 studies. MLD: metachromatic leukodystrophy

Comorbidities and complications related to MLD were reported in a cross-sectional manner in 35 studies [11, 15, 16, 20, 21, 24, 33, 36, 38, 39, 42, 43, 46, 47, 49, 55, 59–61, 64–66, 69, 74, 75, 78, 80, 81, 83, 86, 98–102]. Neuropathy, seizures, gallbladder abnormalities, incontinence, ataxia, epilepsy, and optic atrophy were reported in four or more studies (Fig. 2). Difficulties with swallowing were also reported in four studies, of which three also reported difficulties with breathing, and hypertonia or hypotonia were reported in three studies [15, 16, 21, 61, 81, 101]. Other comorbidities and complications reported (in single studies only) included scoliosis, unilateral left hip subluxation, spastic quadriparesis, dysarthria, visual loss, spasticity, dystonia, abnormal nerve conduction, metabolic acidosis, and decline in language [21, 61, 64, 99]. Additionally, one phenome-wide association study compared four specifical leukodystrophies (X-linked adrenoleukodystrophy, Hurler disease, Krabbe disease, and MLD) in a nationwide pediatric database and found that while developmental delay, epilepsy, fluid and electrolyte disturbances, and respiratory issues were shared morbidities in leukodystrophies, infantile cerebral palsy was uniquely associated with MLD [20].

### Disease progression

Progression-related outcomes in untreated patients with MLD were described in 20 studies containing longitudinal data [5, 9, 39, 42, 45, 49–53, 55–58, 63, 71, 76, 87, 103, 104]. The findings from these studies are summarized in Table 4. Overall, patients with late-infantile MLD were reported to have a faster decline of motor function over time than patients with juvenile MLD (based on data from 10 studies) [9, 39, 53, 55, 56, 58, 61, 63, 76, 103]. Changes in cognitive function in patients with MLD from natural history cohorts were reported in nine studies [5, 45, 51–53, 57, 61, 76, 104], of which one study reported changes over time using the Bayley Scales of Infant Development, second edition [76]. Disease progression in MLD affecting eating and drinking was reported in six studies that included natural history cohorts [5, 39, 42, 49, 53, 55]. Impairment or decline in language was reported in five studies; none of these reported explicitly using the Expressive Language Function Classification in MLD [5, 45, 53, 57, 65].

**Table 4:** Studies reporting progression-related outcomes in untreated (natural history) cohorts

Author(s), year, country	Number of patients with MLD	Association between GMFC-MLD at baseline and progression	Gross motor function	Cognitive function	Eating, drinking, and breathing	Key finding(s)
Artigalas et al. 2010 [9] Brazil	22 late-infantile MLD; 4 juvenile MLD		✔			Reports the age of acquisition and loss of neuropsychomotor developmental milestones in patients with late-infantile and juvenile MLD
Biffi et al. 2008 [76] Italy	16 late-infantile MLD; 9 juvenile MLD		✔	✔		Patients with the 0/0 genotype had the most rapid decline in gross motor function and the most severe cognitive impairment. Patients with the 0/R genotype had progressive and severe motor and cognitive deficit, while patients with the R/R genotype were relatively less impaired, and cognitive function remained stable
Brown et al. 2018 [39] USA	11 juvenile MLD		✔		✔	Caregivers reported impairments in physical functioning related to activities of daily living in patients with juvenile MLD
Chen et al. 2016 [42] Australia	5 juvenile MLD in total; 2 untreated^a^				✔	Describes age (or time after diagnosis) at which patients with juvenile MLD were gastrostomy-fed
Eichler et al. 2016 [81] USA, France, Germany, and Colombia	14 late-infantile MLD; 6 juvenile MLD; 3 adult MLD; 30 caregivers				✔	Across all clinical subtypes of MLD, 50% of caregivers reported that their dependents had difficulty swallowing, and 43% reported breathing/respiratory difficulties
Elgun et al. 2019 [45] Germany	12 sibling pairs (3 late-infantile MLD, 9 juvenile MLD) compared with 61 unrelated children (29 late-infantile MLD, 32 juvenile MLD)	✔		✔		All children in the late-infantile MLD group had rapid and uniform progression of motor function decline In the juvenile MLD group, the course of motor decline was significantly different between siblings and unrelated patients
Fumagalli et al. 2021 [49] Italy	22 late-infantile MLD; 19 juvenile MLD (14 early-juvenile, 5 late-juvenile)	✔			✔	Significant differences in all major disease-related milestones (except death) were observed between the early-juvenile and late-juvenile MLD groups. The late-infantile MLD group displayed earlier loss of trunk control, dysphagia, and death from the time of symptom onset when compared with the early-juvenile group, but loss of ambulation and the start of seizures were similarly rapid between both groups
Groeschel et al. 2011 [51] Germany	33 late-infantile MLD; 35 juvenile MLD	✔		✔		Eichler MLD MRI severity scores were significantly correlated with GMFC-MLD level in both late-infantile and juvenile MLD, but this was less remarkable in the juvenile form
Groeschel et al. 2012 [50] Germany	18 late-infantile MLD	✔				Demyelination load was associated with decline in gross motor function assessed using GMFC-MLD in patients with late-infantile MLD, and was correlated with disease duration
Groeschel et al. 2016 [52] Germany	65 juvenile MLD (24 treated with HSCT; 41 untreated)	✔		✔		Ten years after disease onset, 28 of 41 patients (68%) in the untreated cohort had progressed to GMFC-MLD level 5 and had experienced loss of language, and untreated patients’ Eichler MLD MRI severity scores significantly increased from their early to late disease stages^b^
Harrington et al. 2019 [53] USA	16 late-infantile MLD; 16 juvenile MLD		✔	✔	✔	Caregivers reported that patients with late-infantile MLD experienced rapid disease progression; mean time from first symptom to either complete loss of a gross motor function, a fine motor function, or speech was only 1.0 year (range: 0.2–4.0 years)
Jabbehdari et al. 2015 [55] Iran	12 late-infantile MLD; 6 juvenile MLD		✔		✔	Patients with late-infantile MLD had difficulties with swallowing and eating 2 years after disease onset, and the mental and motor regression process lasted about 10.5 months The progression of juvenile MLD was slower than the late-infantile form
Kehrer et al. 2011 [56] Germany	21 late-infantile MLD; 38 juvenile MLD	✔	✔			Three-quarters of the patients with late-infantile MLD showed their first motor symptoms before the age of 18 months The time to move from GMFC-MLD level 1 to level 6 was significantly longer in patients with juvenile MLD than in patients with late-infantile MLD
Kehrer et al. 2014 [57] Germany	23 late-infantile MLD; 36 juvenile MLD			✔		In the late-infantile MLD group, the median age when language decline occurred was 30 months (range: 17–42 months); almost half (48%) never learned to speak complete sentences. In the juvenile MLD group, the median age when language decline occurred was 8 years There were significant differences between the late-infantile and juvenile MLD groups in terms of problems in concentration and behavioral problems appearing as the first symptoms of disease
Kehre et al. 2021 [5] Germany	35 late-infantile MLD; 56 juvenile MLD (18 early-juvenile, 38 late-juvenile)	✔		✔	✔	For all clinical endpoints, patients with cognitive onset only had significantly slower progression in the course of their disease than patients with either motor onset only or motor and cognitive onset Patients with late-infantile MLD had significantly shorter time from disease onset to their first swallowing difficulties than patients with early-juvenile MLD; however, time from disease onset to tube feeding was similar
Kim et al. 1997 [58] South Korea	7 late-infantile MLD		✔			All children displayed normal development until the onset of symptoms (ranging from 9 to 28 months)
Koto et al. 2021 [16] Japan	15 late-infantile MLD				✔	Most patients (60.0%) had enteral nutrition and 20.0% had a tracheostomy; 6.7% had nasal nutrition
Liaw et al. 2015 [61] Taiwan	5 late-infantile MLD		✔	✔	✔	All children had rapid psychomotor regression after disease onset and became bedridden at a median age of 2 years and 5 months The median age at which language function regressed to “unable to speak” in four patients was 2 years and 6 months, while the median age at which social function deteriorated to “loss of eye contact” in three patients was 3 years and 5 months Of four patients who had data available on support with feeding and breathing, all had a gastric tube fitted (at ages ranging from 2.5 to 3.7 years), and one patient had home BiPAP respiratory support at the age of 5.1 years
Mahmood et al. 2010 [63] USA	98 late-infantile MLD^c^; 78 juvenile MLD^c^		✔			Three triplets with a diagnosis of late-infantile MLD had normal initial development; at approximately 16 months of age, a rapid change in their development was noticed
Martin et al. 2012 [104] France	10 late-infantile MLD; 3 juvenile MLD			✔		In the five late-infantile and three early-juvenile MLD patients with baseline and follow-up data available, changes in mean MRI severity scores over time indicated a more rapid decline in the late-infantile patients
Strolin et al. 2017 [71] Germany	46 juvenile MLD	✔				A significant positive correlation between demyelination load and higher GMFC-MLD level in patients with juvenile MLD was reported
van Rappard et al. 2016 [101] Netherlands	8 late-infantile MLD; 18 juvenile MLD				✔	Most evaluable untreated patients needed feeding via gastrostomy
van Rappard et al. 2018 [87] Netherlands	12 juvenile MLD	✔				A strong correlation between NAA concentration at baseline and GMFC-MLD score at latest follow-up was reported in untreated patients 2 years after diagnosis
Zlotogora et al. 1981 [103] Israel	6 late-infantile MLD		✔			Children with late-infantile MLD showed a significant delay in walking compared with unaffected children

Bold text indicates that outcomes are reported separately for each clinical subtype of MLD. Ticks indicate that the study reported data for the outcome; white boxes indicate that no data were reported for the outcome

BiPAP: bilevel positive airway pressure; GMFC-MLD: Gross Motor Function Classification in MLD; HSCT: hematopoietic stem cell transplantation; MLD: metachromatic leukodystrophy; MRI: magnetic resonance imaging; NAA: *N*-acetylaspartate; SLR: systematic literature review

^a^Untreated patients were described as a reference or control for patients receiving treatment

^b^Kaplan–Meier survival plots for both treated and untreated patients with GMFC-MLD level < 5 and without loss of language, and Eichler MLD MRI severity scores for both treated and untreated patients are reported in Figs. 3 and 4 of the publication but were not extracted for this SLR

^c^SLR publication

Changes in GMFC-MLD scores over time were reported in nine studies that included untreated patients [5, 45, 49–52, 56, 71, 87]. Generally, children with late-infantile MLD had more rapid deterioration of gross motor function than children with juvenile-onset MLD. The age at entry into a certain GMFC-MLD category was reported to be more uniform in late-infantile than juvenile MLD [45, 56]. There was also some evidence of a positive correlation between demyelination load and GMFC-MLD scores [50, 51, 71].

Gross motor function (not assessed in relation to baseline GMFC-MLD) was reported in 10 studies that included untreated patients [9, 39, 53, 55, 56, 58, 61, 63, 76, 103]. Time of acquisition and loss of neuropsychomotor milestones were reported in one study [9]. Overall, these studies showed that patients with late-infantile MLD had faster declines of gross motor function over time than patients with juvenile MLD.

### Mortality and survival

Data on mortality for untreated patients were reported in seven studies [10, 22, 49, 52, 61, 63, 101]. Mortality over time was reported in five studies [49, 52, 61, 63, 101], and the findings from these studies are summarized in Table 5. Survival measured from birth was reported in three studies [49, 61, 63], and survival measured from the onset of first symptoms was reported in three studies, of which two reported on mortality by clinical subtype [49, 52, 63]. Fumagalli et al. reported the 10-year survival rates from symptom onset for late-infantile MLD (40%) and early-juvenile MLD (80%), and found that survival rates were lower in patients with late-infantile MLD than in patients with juvenile MLD [49]. Van Rappard et al. reported that 8/22 untreated patients (36%) died 22–72 months after diagnosis [101]. Mahmood et al. found that, since 1921, the 10-year survival rates from symptom onset were 0%, 44.3%, and 69.6% for late-infantile, juvenile, and adult MLD, respectively. Further analysis by decades indicated increased survival over time for all types of MLD. Specifically, 5-year survival reported after 1990 was significantly higher than that reported prior to 1970 for all subtypes of MLD (late-infantile: 52% vs. 14%; juvenile: 100% vs. 46%; adult: 95% vs. 67%). The systematic review described by Mahmood et al. excluded studies that only included patients receiving transplants [63]. Mortality according to genetic variants was reported for a Saudi Arabian cohort through a 5-year time frame. In this study, 7/11 children with variants in *ARSA* had died, whereas 10/10 patients with *PSAP* variants were alive [22].

**Table 5:** Mortality over time and overall survival in untreated children with MLD

Author(s), year, country	Late-infantile MLD		Juvenile MLD	
	OS for late-infantile MLD, years	Survival rates for late-infantile MLD	OS for juvenile MLD, years	Survival rates for juvenile MLD
Fumagalli et al. 2021 [49] Italy	Median OS (time from symptom onset) • Overall (*n* = 22): 8.42^a^ • Ambulant (*n* = 13): 9.17 • Non-ambulant (*n* = 9): 8.42 (*P* = 0.9424 vs. ambulant) • Median OS (from birth): • Ambulant (*n* = 13): 10.17 • Non-ambulant (*n* = 9): 9.42 (*P* = 0.5768 vs. ambulant)	• 5 years from symptom onset: 56% • 10 years from symptom onset: 40%	Median OS (time from symptom onset) • Early-juvenile (*n* = 14): not reached • Early-juvenile subgroup with motor symptoms at onset (*n* = 9): not reached • Early-juvenile subgroup with only cognitive symptoms at onset (*n* = 5): not reached • Late-juvenile (*n* = 5): not reached	Early-juvenile (*n* = 14) • 5 years: 90% •10 years: 80% •15 years: 68.6% Early-juvenile subgroup with motor symptoms at onset (*n* = 9) •15 years: 68.6% Early-juvenile subgroup with only cognitive symptoms at onset (*n* = 5) • 15 years: 66.7% Late-juvenile (*n* = 5) • 5 years: 100% • 10 years: 100% •15 years: 100%
Groeschel et al. 2016 [52] Germany	N/A	N/A	Untreated •Median age at follow-up: 15.8 years (range: 3.9–47.1 years)	Untreated • 5 years from disease onset:^b^ 100% (41/41) • 5 years from baseline evaluation:^b^ 73% (30/41)
Liaw et al. 2015 [61] Taiwan	Of 5 patients in total, 3 patients had follow-up data: 2 died at 7.34 years old and 4.75 years old (both from respiratory failure) and 1 was alive at 8 years of age	NR	N/A	N/A
Mahmood et al. 2010 [63] USA	NR	5 years from onset of symptoms: 25.1% 10 years from onset of symptoms: 0%^c^	NR	5 years from onset of symptoms: 70.3% 10 years from onset of symptoms: 44.3%^c^

Author(s), year, country	Studies not reporting by subtype of MLD	
	OS for MLD, years	Survival rates for MLD, years
van Rappard et al. 2016 [101] Netherlands	8 untreated patients (36%) died 22–72 months after diagnosis^d^	At latest assessment Untreated (*n* = 22): 63.6% at ~ 70 months^e^ since diagnosis^f^

No studies were identified reporting mortality data for untreated adult patients with MLD

CI: confidence interval; HR: hazard ratio; MLD: metachromatic leukodystrophy; N/A: not applicable; NR: not reported; OS: overall survival

^a^The insertion of a percutaneous gastrostomy tube was not associated with prolonged survival in patients with late-infantile MLD (HR 0.78 [95% CI: 0.33–1.83]; *P* = 0.572)

^b^Median (range) age at disease onset was 6.5 (2.7–16.0) years

^c^The systematic review described in Mahmood et al. 2010 excluded studies that were limited to patients receiving transplants

^d^Subtypes NR; all were < 16 years of age

^e^Approximate values read from graphs in publication

^f^Subtypes NR

### Humanistic evidence

Four studies reported the impact of MLD on patients and caregivers, which are later described in detail [77, 81, 85, 105]. Pang et al. reported data on quality of life (QoL) for 21 patients with MLD and their caregivers. Caregivers in Germany, the UK, and the USA were asked to complete a Pediatric Quality of Life Inventory and an EuroQoL 5-dimension (EQ-5D) questionnaire. Most caregivers (71%) self-reported problems with anxiety/depression, and the mean EQ-5D index values were lower than those for the population norms for each of the three respective countries [77].

Ammann-Schnell et al. asked the parents and families of children with MLD (8 late-infantile and 21 juvenile-onset) about the impact of MLD on their QoL and general family functioning. All reported significantly lower health-related QoL (HRQoL) than the parents and families of unaffected children (*P* < 0.001), with mothers being more significantly affected than fathers (*P* < 0.05). Parents of children with late-infantile MLD reported worse HRQoL and family functioning than parents of children with the juvenile form of MLD, and scores worsened with increasing time from diagnosis and as children reached an advanced, terminal disease stage [85].

Eichler et al. conducted caregiver interviews to identify the specific clinical and QoL outcomes relevant for both patients with MLD and their caregivers. Caregivers reported that the most troublesome symptoms for them were immobility (9/30 caregivers) and respiratory difficulties (6/30), across all MLD subtypes (late-infantile, juvenile, and adult); however, patients reported that the most troublesome symptom for them was difficulty with communication (6/30), according to their caregivers. Caregivers reported considerable emotional burden, most commonly caused by the need to be confined to the home to provide care (16/30), relationship difficulties with spouses (6/30), feelings of fear (11/30), and depression or worry (8/30 each). Patients most commonly reported loss of autonomy (13/30) and their limited relationships with peers (9/30) as the most emotionally troublesome effects of their condition [81].

Pang et al. (2021) reported health state utility values for patients with infantile and juvenile MLD in the UK, developed through literature review and interviews with clinicians (*n* = 6) and caregivers (*n* = 21). Health states were defined by GMFC-MLD levels 0–6 and by Development Quotient scores for three cognitive functioning levels (normal/mild, moderate, and severe) for patients with juvenile MLD; late-infantile health states were defined by GMFC-MLD only. Clinicians reported that, from GMFC-MLD level ≥ 2, patients experienced significant symptoms, with significant overlap between levels, from level 2 to level 6. Reported symptoms included problems with swallowing, muscle spasms, digestive issues, seizures, and sleep. Health states were valued by 101 members of the UK general public via visual analog scale and time trade-off (TTO) assessment, including the lead-time method. Lead-time TTO is a method whereby individuals express their preferences for different health states by hypothetically trading between QoL and quantity of life, without having to consider whether these states are better or worse than being dead [106]. For late-infantile MLD health states, mean TTO values ranged from 0.71 for GMFC-MLD level 1 to − 0.47 for GMFC-MLD 

level 6. Utility values were lower for juvenile health states than for late-infantile health states and worsened with cognitive status: in the normal/mild cognitive group, mean utility values ranged from 0.90 for GMFC-MLD level 1 to − 0.07 for GMFC-MLD level 4; in the moderate cognitive group, mean scores ranged from 0.85 for GMFC-MLD level 0 to − 0.62 for GMFC-MLD level 6; and in the severe group, mean scores ranged from 0.37 for GMFC-MLD level 0 to − 0.70 for GMFC-MLD level 5 [105].

### Economic evidence

Two studies reported resource use for untreated patients with MLD [81, 82]. One study reported healthcare resource use post-MLD diagnosis for 24 patients with MLD (12 late-infantile and 12 juvenile) in the UK. 

Overall, these patients had a mean of 3.7 (standard deviation [SD] 7.2) outpatient hospital attendances per patient-year (4.0 [SD 9.9] for patients with late-infantile MLD; 3.4 [SD 3.1] for patients with juvenile MLD). Mean elective inpatient admissions per patient-year were 2.6 (SD 8.8) for patients with late-infantile MLD and 0.1 (SD 0.2) for patients with juvenile MLD (1.4 [SD 6.2] per patient-year overall). Overall, patients with MLD had a mean of 0.05 (SD 0.10) day case admissions per patient-year [82]. In another study of patients from Colombia, France, Germany, and the USA, 12/22 caregivers interviewed reported that their respective patients required an average of 1–3 visits to primary care practitioners per month, and nine reported an average of 1–3 specialist visits per month. Eight patients had required at least 11 hospitalizations since diagnosis; however, given that the two studies used different measures of economic impacts, these results cannot be directly compared [81].

## Discussion

This SLR offers a comprehensive and robust analysis of topics related to the natural history and burden of illness of MLD, having been designed and conducted using methodology in accordance with the 2020 PRISMA guidelines. To our knowledge, it is the first SLR to provide a broad overview of these areas, complementing a previous SLR that summarized mortality data in studies of MLD from 1920 to June 30, 2006 [63]. The data reported show that the disease course varies widely between patients with MLD and that patients exhibit a wide range of signs, symptoms, comorbidities, and complications. These data also provide an important comparator dataset for outcomes when evaluating the effects of new disease-modifying therapies for MLD.

The wide variation in age at diagnosis and time to diagnosis reported in this SLR is likely to be due to differences in diagnostic methods over time and between countries; it also highlights the unmet need for early diagnosis of patients with MLD. Increased disease awareness and more frequent and widespread diagnosis of presymptomatic patients through newborn screening would help to reduce the wide variation in age at diagnosis and time to diagnosis for this disease. Crucially, newborn screening for MLD has been shown to be possible in a real-world scenario and to have a high degree of support among caregivers of patients with MLD, although a number of challenges to its implementation remain [107–111]. Various prospective screening pilots are ongoing, and between October 2021 and August 2022, a program in Germany screened ~ 50,000 babies and identified four *ARSA* heterozygotes, one of whom was MLD positive [112]. Improved availability of newborn screening would also help to increase the proportion of patients who are eligible for gene therapy [113–115].

Patients with MLD experience a high disease burden that increases as the disease progresses, with commonly reported comorbidities and complications including seizures, ataxia, and optic atrophy. Although patients with MLD typically die prematurely, data from a previous SLR suggest that survival has improved over time for all clinical subtypes of MLD [63]. Currently, little evidence is available on time to loss of motor function, although this may be a more useful measure than mortality for understanding disease progression and its subsequent impact on QoL.

Typically, patients with late-infantile MLD have earlier onset, a more rapid decline of motor function over time, higher GMFC-MLD levels, and lower survival than patients with juvenile MLD. Patients with late-infantile MLD typically present predominantly with motor function symptoms and delays in reaching developmental milestones, whereas patients with juvenile MLD usually present with motor, cognitive, and behavioral symptoms. In the initial presentation of the late-juvenile and adult subtypes, cognitive symptoms predominate. These findings are generally consistent with the clinical presentation of patients with different MLD subtypes in a recent study [116]. However, in this study only a small proportion of patients with late-juvenile MLD (1/12, 8%) had a cognitive-only phenotype, suggesting that the clinical phenotype of patients with late-juvenile MLD varies widely [116]. In a study of descriptions given by caregivers of children with MLD (20 late-infantile, 11 juvenile, 1 borderline late-infantile/juvenile), coordination difficulties, clonus/tremors, and comprehension challenges were identified as the most common initial signs and symptoms of MLD, supporting their frequent documentation in this SLR [117]. The wide variation in signs, symptoms, and disease progression among patients with MLD, combined with the need for early diagnosis to facilitate prompt treatment initiation, mean that there is a need to be able to predict clinical subtype and disease progression, which some studies have begun to investigate [118, 119]. For example, data from a recent study suggest that early developmental delay can precede neurologic regression in patients with late-infantile MLD [120].

Available evidence shows that MLD has a clear impact on the QoL of patients and families, with patients being particularly affected by loss of autonomy and limited relationships with peers, and that caregivers were found to report high levels of anxiety and depression [77, 81]. Supporting this, in a recent study of caregivers of patients with late-infantile MLD, most felt that delaying the decline in gross motor function would have a meaningful impact on patients [121]. Overall, these findings highlight the need for psychological support for patients with MLD and their families.

Key knowledge gaps include data on mortality by clinical subtype, humanistic and economic outcomes, and differences between the early- and late-juvenile MLD subtypes. Since this SLR was conducted, an additional study on the impact of MLD on caregivers has been published [122]. In this study, the EQ-5D questionnaire was administered to caregivers of patients with MLD in Belgium, France, Germany, Norway, and the USA. In line with the findings from Pang et al. [77], caregivers had EQ-5D values below national population norms and reported high levels of anxiety/depression. Differences between caregivers of patients with late-infantile MLD compared with those of patients with juvenile MLD were also observed; the former group was more likely to report a negative impact on familial relationships, and the latter group reported more lifestyle changes and dissatisfaction with their personal lives [122].

In addition to the knowledge gaps described earlier, there were also insufficient epidemiological data to compare incidence and prevalence by country or region, in part owing to varying methodology and study periods between publications. Data on disease course, such as reports of signs and symptoms, were mainly qualitative, limiting the collation of data across studies. Improved reporting of quantitative data on signs and symptoms, such as measurements of ASA activity or biomarker levels, could help to facilitate predictions of the disease course and inform treatment decisions [118, 119].

The studies included in this SLR covered a broad geographical evidence base, and a substantial number of studies reported detailed baseline characteristics and outcomes separately for the different clinical subtypes of MLD. Quality assessment was performed using the ROBINS-I tool for non-randomized interventional clinical studies and informally for non-interventional studies. Limitations include those common among SLRs, such as the possibility that some relevant studies may not have been detected by the searches if they did not mention any terms of interest in their titles or abstracts; this limitation results from balancing the need to compromise between identifying all relevant evidence and limiting searches so that the scope of the review remains focused and manageable. Another limitation was that data in figures without detailed labeling could not be extracted in full, meaning that, for these, only trends could be determined.

MLD is a devastating disease that shortens life and reduces QoL, especially as the disease progresses. Further studies on clinical, humanistic, and economic outcomes, particularly by clinical subtype, will help to inform healthcare decisions for patients with MLD.

### Supplementary Information

**Additional file 1.** Search strings for Embase (1974–2021 [search run on June 23, 2021]).

**Additional file 2.** Search strings for Ovid MEDLINE (1946–2021 [search run on June 23, 2021]).

**Additional file 3.** Search strings for Cochrane Library (search run on 23 June 2021).

## Data Availability

Data sharing not applicable because no new data were generated.

## Abbreviations

AE: Adverse event
ANA: American ARSA, arylsulfatase A gene
ASA: Arylsulfatase A
CI: Confidence interval
CRD: Centre for Reviews and Dissemination
CSF: Cerebrospinal fluid
ELFC-MLD: Expressive Language Function Classification in MLD
EQ-5D: EuroQol 5-dimension questionnaire
GMFC-MLD: Gross Motor Function Classification in MLD
GMFM-88: 88-Item Gross Motor Function Measure
HRQoL: Health-related QoL
HSCT: Hematopoietic stem cell transplant
LQLA: Leukodystrophy Quality of Life Assessment
LSD: Lysosomal storage disease
MLD: Metachromatic leukodystrophy
MRI: Magnetic resonance imaging
MRS: Magnetic resonance spectroscopy
PedsQL: Pediatric Quality of Life Inventory
PICOS: Participants, Interventions, Comparators, Outcomes, and Study design
PRISMA: Preferred Reporting Items for Systematic review and Meta-Analysis
PSAP: Prosaposin
QoL: Quality of life
RCT: Randomized controlled trial
ROBINS-I: Risk Of Bias In Non-randomized Studies - of Interventions
SAPB: Sphingolipid activator protein B
SD: Standard deviation
SLR: Systematic literature review
TEAE: Treatment-emergent adverse event
TTO: Time trade-off
