# Supplementary material for: The natural history and burden of illness of metachromatic leukodystrophy: a systematic literature review
Source: Eur J Med Res. 2024 Mar 18;29:181. doi: 10.1186/s40001-024-01771-1 (PMC10946116; doi:10.1186/s40001-024-01771-1)
Supplement: Supplementary file 1 — Additional file 1. Search strings for Embase (1974–2021 [search run on June 23, 2021]). [file 40001_2024_1771_MOESM1_ESM.docx]

**Additional file 1** Search strings for Embase (1974–2021 [search run on June 23, 2021])

| **#** | **Searches** | **Results** |
| --- | --- | --- |
| 1 | exp metachromatic leukodystrophy/ | 2134 |
| 2 | metachromatic leukodystroph$.mp. | 2331 |
| 3 | (Arylsulfatase A deficiency or ASA deficiency or sulphatide lipidosis or sulfatide lipidosis or sulphatidosis or sulfatidosis or Greenfield's disease or metachromatic leukoencephalopathy or cerebroside sulfatase deficiency disease or (cerebral sclerosis and metachromatic)).mp. | 126 |
| 4 | 1 or 2 or 3 | 2342 |
| 5 | Clinical trial/ | 1,006,754 |
| 6 | Randomized Controlled Trial/ | 663,043 |
| 7 | controlled clinical trial/ | 464,260 |
| 8 | multicenter study/ | 291,311 |
| 9 | Phase 1 clinical trial/ | 58,218 |
| 10 | Phase 2 clinical trial/ | 88,468 |
| 11 | Phase 3 clinical trial/ | 53,766 |
| 12 | Phase 4 clinical trial/ | 4346 |
| 13 | exp RANDOMIZATION/ | 91,351 |
| 14 | Single blind procedure/ | 43,016 |
| 15 | Double blind procedure/ | 185,377 |
| 16 | Crossover procedure/ | 67,415 |
| 17 | PLACEBO/ | 368,216 |
| 18 | randomi?ed controlled trial$.tw. | 260,724 |
| 19 | rct.tw. | 42,470 |
| 20 | (random$ adj2 allocat$).tw. | 46,950 |
| 21 | single blind$.tw. | 27,175 |
| 22 | double blind$.tw. | 221,195 |
| 23 | ((treble or triple) adj blind$).tw. | 1381 |
| 24 | placebo$.tw. | 327,863 |
| 25 | Prospective Study/ | 693,325 |
| 26 | (single arm or single-arm or noncomparative or non-comparative).tw. | 25,618 |
| 27 | exp Meta Analysis/ | 219,444 |
| 28 | ((meta adj analy$) or metaanalys$).tw. | 266,539 |
| 29 | (systematic adj (review$1 or overview$1)).tw. | 256,340 |
| 30 | ((indirect or indirect treatment or mixed-treatment) adj comparison*).ti,ab,kw. | 4499 |
| 31 | (comparative adj3 (efficacy or effectiveness)).ti,ab,kw. | 21,660 |
| 32 | (outcomes research or relative effectiveness).ti,ab,kw. | 14,471 |
| 33 | or/5-32 | 2,885,504 |
| 34 | Clinical study/ | 155,826 |
| 35 | Case control study/ | 174,061 |
| 36 | Family study/ | 25,360 |
| 37 | Longitudinal study/ | 157,289 |
| 38 | Retrospective study/ | 1,092,956 |
| 39 | Prospective study/ | 693,325 |
| 40 | Randomized controlled trials/ | 205,795 |
| 41 | 39 not 40 | 685,473 |
| 42 | Cohort analysis/ | 720,549 |
| 43 | (Cohort adj (study or studies)).mp. | 355,452 |
| 44 | (Case control adj (study or studies)).tw. | 144,831 |
| 45 | (follow up adj (study or studies)).tw. | 66,458 |
| 46 | (observational adj (study or studies)).tw. | 193,062 |
| 47 | (epidemiologic$ adj (study or studies)).tw. | 111,713 |
| 48 | (cross sectional adj (study or studies)).tw. | 254,989 |
| 49 | (registry or register$ or survey).ti,ab. | 1,188,796 |
| 50 | (real world or RWE).ti,ab. | 86,683 |
| 51 | Real-life.ti,ab. | 37,318 |
| 52 | or/34-38,41-51 | 4,091,116 |
| 53 | case series/ or case series.mp. | 148,990 |
| 54 | ((economic or pharmacoeconomic) adj1 (evaluation or assessment or analys?s or stud*)).mp. | 38,691 |
| 55 | Cost effectiveness analysis/ | 160,171 |
| 56 | Cost minimization analysis/ | 3650 |
| 57 | Cost benefit analysis/ | 87,451 |
| 58 | Cost utility analysis/ | 10,443 |
| 59 | Budget impact/ | 7 |
| 60 | Cost consequence analysis/ | 23 |
| 61 | (CEA or CMA or CBA or CUA or CCA).mp. | 75,542 |
| 62 | "health care cost"/ or "drug cost"/ or "hospital cost"/ or "hospitalization cost"/ or "nursing cost"/ | 287,241 |
| 63 | ((health or global) adj2 burden).mp. | 26,444 |
| 64 | ((direct or indirect or societ* or employe*) adj2 (resource* or benefit*)).mp. | 8316 |
| 65 | exp caregiver burden/ or exp caregiver support/ | 11,482 |
| 66 | (caregiver* or carer*).mp. | 143,243 |
| 67 | economics/ | 241,438 |
| 68 | budget*.mp. | 54,770 |
| 69 | cost*.mp. | 1,153,085 |
| 70 | productivity/ or productivity.mp. | 95,695 |
| 71 | absenteeism.mp. or absenteeism/ | 20,733 |
| 72 | "length of stay"/ | 211,141 |
| 73 | Cost control/ | 70,809 |
| 74 | (fiscal or financ* or funding).mp. | 366,829 |
| 75 | financial management.mp. or financial management/ | 116,018 |
| 76 | health care utilization/ or health care utili*.mp. | 81,355 |
| 77 | health care financing.mp. or health care financing/ | 15,595 |
| 78 | health economics.mp. or health economics/ | 38,143 |
| 79 | (burden adj2 (illness or disease$ or treatment*)).mp. | 68,685 |
| 80 | resource allocation/ | 22,214 |
| 81 | exp resource management/ | 41,347 |
| 82 | budget/ | 30,605 |
| 83 | pharmacoeconomics/ or pharmacoeconomic*.mp. | 92,582 |
| 84 | pay?r.mp. | 18,428 |
| 85 | health care planning.mp. or health care planning/ | 101,890 |
| 86 | (resource adj2 (use* or utili?ation or allocat* or burden or health)).mp. | 70,869 |
| 87 | (economic adj5 (burden or impact)).mp. | 42,485 |
| 88 | cost of illness.mp. or "cost of illness"/ | 21,454 |
| 89 | cost control.mp. or "cost control"/ | 71,773 |
| 90 | Economics, Medical/ | 32,561 |
| 91 | Quality-Adjusted Life years/ | 29,154 |
| 92 | (quality adjusted or adjusted life year$).ti,ab,kw. | 27,651 |
| 93 | (qaly$ or qald$ or qale$ or qtime$).ti,ab,kw. | 22,465 |
| 94 | (illness state$1 or health state$1).ti,ab,kw. | 12,358 |
| 95 | (hui or hui1 or hui2 or hui3).ti,ab,kw. | 2545 |
| 96 | (multiattribute$ or multi attribute$).ti,ab,kw. | 1280 |
| 97 | (utility adj3 (score$1 or valu$ or health$ or cost$ or measur$ or disease$ or mean or gain or gains or index$)).ti,ab,kw. | 26,752 |
| 98 | utilities.ti,ab,kw. | 12,837 |
| 99 | (eq-5d or eq5d or eq-5 or eq5 or euro qual or euroqual or euro qual5d or euroqual5d or euro qol or euroqol or euro qol5d or euroqol5d or euro quol or euroquol or euro quol5d or euroquol5d or eur qol or eurqol or eur qol5d or eur qol5d or eur?qul or eur?qul5d or euro$ quality of life or european qol).ti,ab,kw. | 24,296 |
| 100 | (euro$ adj3 (5 d or 5d or 5 dimension$ or 5dimension$ or 5 domain$ or 5domain$)).ti,ab,kw. | 7105 |
| 101 | (sf36$ or sf 36$ or sf thirtysix or sf thirty six).ti,ab,kw. | 40,707 |
| 102 | (time trade off$1 or time tradeoff$1 or tto or timetradeoff$1).ti,ab,kw. | 3006 |
| 103 | quality of life/ and ((quality of life or qol) adj (score$1 or measure$1)).ti,ab,kw. | 27,789 |
| 104 | quality of life/ and ec.fs. | 46,839 |
| 105 | quality of life/ and (health adj3 status).ti,ab,kw. | 17,643 |
| 106 | (quality of life or qol).ti,ab,kw. and Cost-Benefit Analysis/ | 5912 |
| 107 | or/54-106 | 2,399,503 |
| 108 | 4 and (33 or 52 or 53 or 107) | 371 |
| 109 | (animal$ not human$).sh,hw. | 4,509,490 |
| 110 | 108 not 109 | 370 |
| 111 | limit 110 to english language | 354 |
| 112 | remove duplicates from 111 | 348 |
